# Dopaminergic and cholinergic modulation of the amygdala is altered in female mice with oestrogen receptor β deprivation

**DOI:** 10.1038/s41598-023-28069-2

**Published:** 2023-01-17

**Authors:** Daniel Kalinowski, Krystyna Bogus-Nowakowska, Anna Kozłowska, Maciej Równiak

**Affiliations:** 1 Department of Animal Anatomy and Physiology, Faculty of Biology and Biotechnology, University of Warmia and Mazury in Olsztyn, pl. Łódzki 3, 10-727 Olsztyn, Poland; 2 Department of Human Physiology and Pathophysiology, School of Medicine, University of Warmia and Mazury in Olsztyn, Warszawska 30, 10-082 Olsztyn, Poland

**Keywords:** Neuroscience, Cellular neuroscience, Diseases of the nervous system, Emotion

## Abstract

The amygdala is modulated by dopaminergic and cholinergic neurotransmission, and this modulation is altered in mood disorders. Therefore, this study was designed to evaluate the presence/absence of quantitative alterations in the expression of main dopaminergic and cholinergic markers in the amygdala of mice with oestrogen receptor β (ERβ) knock-out which exhibit increased anxiety, using immunohistochemistry and quantitative methods. Such alterations could either contribute to increased anxiety or be a compensatory mechanism for reducing anxiety. The results show that among dopaminergic markers, the expression of tyrosine hydroxylase (TH), dopamine transporter (DAT) and dopamine D_2_-like receptor (DA_2_) is significantly elevated in the amygdala of mice with ERβ deprivation when compared to matched controls, whereas the content of dopamine D_1_-like receptor (DA_1_) is not altered by ERβ knock-out. In the case of cholinergic markers, muscarinic acetylcholine type 1 receptor (AChR_M1_) and alpha-7 nicotinic acetylcholine receptor (AChR_α7_) display overexpression while the content of acetylcholinesterase (AChE) and vesicular acetylcholine transporter (VAChT) remains unchanged. In conclusion, in the amygdala of ERβ knock-out female the dopaminergic and cholinergic signalling is altered, however, to determine the exact role of ERβ in the anxiety-related behaviour further studies are required.

## Introduction

Sex hormones have a considerable influence on brain functioning and behaviour. There is a strong relationship between low oestrogen levels and emotional disorders such as mood swings, anxiety, and depression in postmenopausal women^1,2^, whereas oestrogen replacement therapy reduces these symptoms^3^. Many reports also indicate that the risk of anxiety and depression is higher in perimenopausal women compared to pre- or postmenopausal women even when considering factors such as age, race, and lifestyle among others^4,5^. Finally, in rodents, reduced oestrogen signalling due to oestrogen receptor β (ERβ) knock-out increases anxiety in females (but not in males)^6,7^, while ERβ agonists usually induce severe anxiolytic actions^8^. It is generally known that one of the most important structure responsible for overall regulation of emotional behaviour, especially in the processing of fear and anxiety is the amygdala^9,10^ and oestrogens reduce neuronal excitability in this brain structure^11–13^ through their receptors^14,15^, thereby reduced oestrogen signalling as a result of ERβ knock-out is anxiogenic. It was noted that the function of this brain structure is always impaired in anxiety disorders^16^. This may be associated with changes in neurotransmission that were well-documented in many reports^17–19^.

Furthermore, electrophysiological studies revealed that in mutant mice enhanced anxiety coincides with a reduced threshold for induction of synaptic plasticity in the medial amygdala which may result in excessive responses to naturally harmless stimuli, a hallmark of fear and anxiety disorders^7^. Finally, pharmacological manipulation of γ-aminobutyric acid (GABA) type A (GABA_A_) receptors in the amygdala of wild-type mice mimics the consequences of ERβ knock-out^7^ suggesting involvement of the inhibitory neurotransmitter GABA in this phenomenon. Indeed, it is plausible that abnormal GABA activity may account for the increased anxiety in ERβ knock-out mice^20^ as it is associated with fear and anxiety as evidenced by various experiments^21–23^. Furthermore, recent evidence has shown that calbindin expression, a marker of the largest GABAergic subpopulation, is strongly reduced in the amygdala of these mice^24^ and this decrease has a potential to reduce interneuron firing and threshold for synaptic plasticity^25,26^. Indeed, oestrogen interacts with neuromodulatory systems including dopaminergic and cholinergic systems^20,27–29^, which are involved in controlling fundamental behaviours by interacting with broad areas of the brain^30,31^. These systems project to many areas, including the cortex, forebrain, striatum, hippocampus, and also amygdala^32–36^.

However, to the best of our knowledge, there are no publications to date explaining the relationship between oestrogen and other neurotransmitter systems e.g., dopaminergic, and cholinergic. This is extremely important because the amygdala is densely packed with dopaminergic innervation from the ventral tegmental area and substantia nigra^37^. It is known that dopamine (DA) release increases in the basolateral amygdala under stressful conditions, and an activation of dopamine receptors (DAs) in this region is crucial to generate fear-related memory^38–40^. Furthermore, some reports have pointed to a role for the DA in anxiety^41,42^. However, the action of DA on the amygdala circuits is rather unclear and depends on the specific populations of innervated neurons and receptors used. Generally, DAs have been classified in two types, D_1_-like receptors (DA_1_) and D_2_-like receptors (DA_2_)^43^. The activation of DA_1_ increases neuronal activity whereas activation of DA_2_ restrains neuronal activity^44,45^, and the interaction between DA_1_ and DA_2_ is necessary to express most dopaminergic-related behaviours^46,47^, e.g., DA_1_ and DA_2_ are important in mediating anxiety, especially visible in an elevated plus maze test^48,49^. It should be emphasized that in natural conditions, oestrogens strongly modulate dopaminergic neurotransmission. It may increase or decrease DA activity e.g., by its degradation, reuptake, and recover, also by upregulating dopaminergic receptors^27^ or by reducing the affinity of the DA transporter (DAT)^50^. Furthermore, many of the effects, have been shown to be mediated by ERβ signalling^51–55^. It is well-known that the amygdala also receives a dense cholinergic innervation from the ventral pallidum and substantia innominata of the basal forebrain^56^. Muscarinic receptors (AChR_M_) can inhibit or excite postsynaptic neurons^57^, whereas nicotinic receptors (AChR_n_) directly excite postsynaptic neurons^58^. The acetylcholine (ACh) releases in the amygdala plays a role in anxiety disorders^59^. Previous studies in rodents have shown an increase in anxiety, specifically in depressed mood and co-morbid anxiety states, as a result of increased AChR_n_ activity^60^. Especially, α7 antagonist infusion or knock-down has been associated to an anxiolytic behaviour as a conclusion of light–dark box and tail suspension test^61^, and high concentrations of this receptor in the amygdala can play a significant role in the modulation of changes in amygdala function^62^. On the other hand, AChR_M1_ seems to be significant in fear extinction because direct infusion of oxotremerine (the muscarinic agonist) into the basolateral amygdala induces an enhancement in fear extinction learning^63^. Notably, oestrogen has also been demonstrated to shape cholinergic neurotransmission in the brain, often via ERβ^64,65^. The oestrogen increases choline and choline acetyltransferase reuptake in the hippocampus and frontal lobe^66^, but no data are available for the amygdala.

Altogether, the amygdala is strongly modulated by dopaminergic and cholinergic fibres, which activate both glutamatergic and GABAergic neurons and thereby have a considerable influence on the amygdala inhibitory/excitatory balance and processing. Further, in normal conditions dopaminergic and cholinergic neurotransmission is heavily modulated by oestrogen-signalling, so this modulation could be altered in ERβ knock-out mice due to ERβ deficiency. To test this hypothesis, a single immunofluorescent staining was performed to assess the presence/absence of quantitative changes in the expression of tyrosine hydroxylase (TH), DAT, DA_1_, DA_2_, acetylcholinesterase (AChE), vesicular acetylcholine transporter (VAChT), AChR_M1_ and AChR_α7_ in the amygdala of ERβ knock out female mice. To make the text easier to read, the immunoreactive elements (somata, fibres and neuropil) expressing these markers will be uniformly described as TH+, DAT+, DA_1_+, DA_2_+, AChE+, VAChT+, AChR_M1_+ and AChR_α7_+, respectively. Additionally, the hierarchical clustering and functional importance of mutual interactions of these proteins was also studied using Gene Ontology^67^.

## Results

The results of this present study show that the expression of the main dopaminergic markers, including TH, DAT and DA_2_, is significantly elevated in the amygdala of ERβ knock-out mice compared to matched controls, and the only exception constitutes DA_1_ which is not affected by ERβ loss. Furthermore, although the expression of cholinergic markers such as AChE and VAChT does not differ in ERβ knock-out mice compared to matched controls, the density of cholinergic receptors such as AChR_M1_ and AChR_α7_ is increased in mutant mice.

### Dopaminergic markers in the amygdala of ERβ knock-out mice

**Figure 1:**
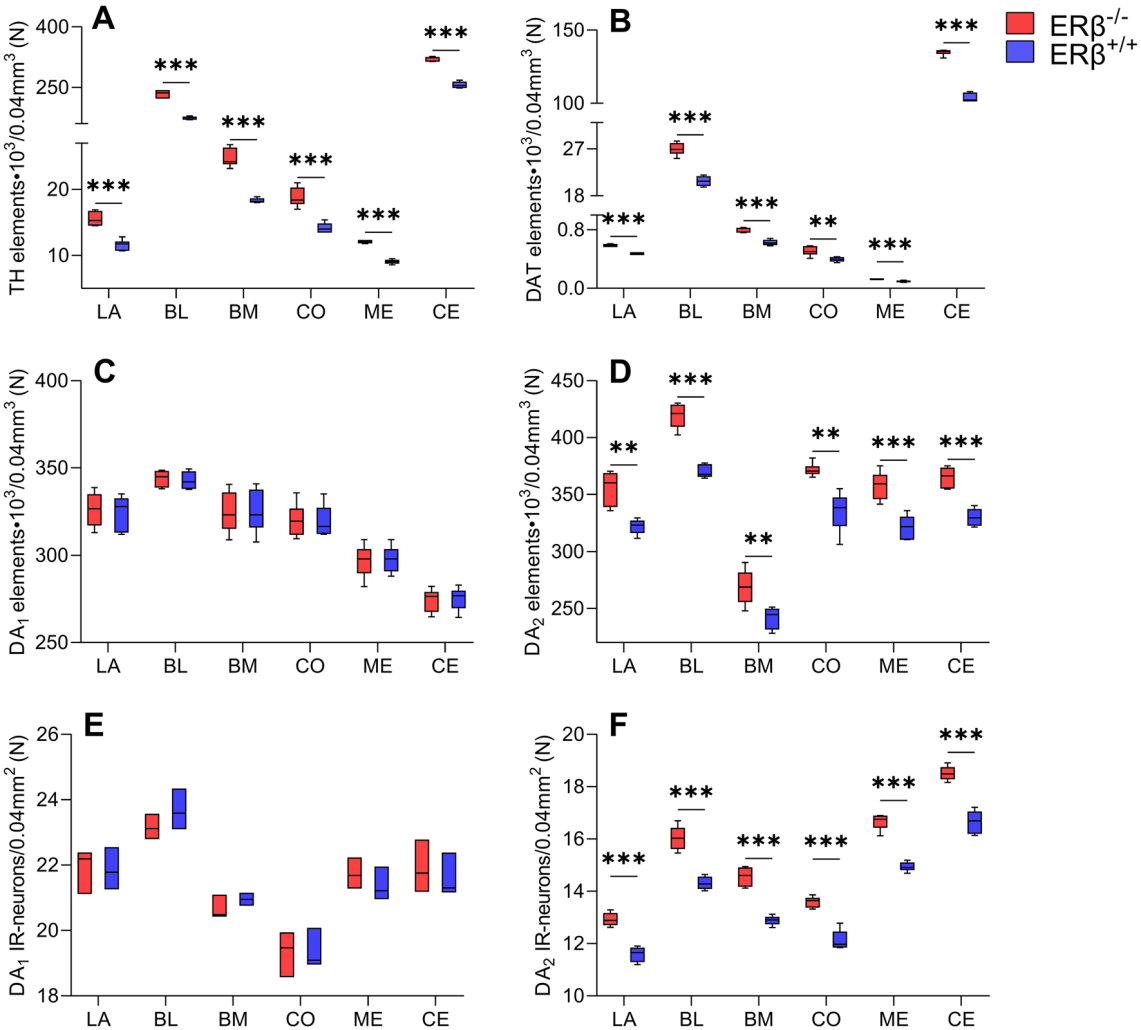
The densitometry of dopaminergic markers such as tyrosine hydroxylase (TH), dopamine transporter (DAT), dopamine D_1_-like receptor (DA_1_) and dopamine D_2_-like receptor (DA_2_) in the amygdala of ERβ knock-out (ERβ^−/−^) and wild-type (ERβ^+/+^) mice, *n* = 6 per group. Note that the volume density of TH (**A**), DAT (**B**) is significantly elevated in ERβ^−/−^ mice when compared to ERβ^+/+^ mice whereas values for DA_1_ (**C**) do not differ in both mice lines but for DA_2_ (**D**) is significantly elevated in ERβ^−/−^ mice. Additionally, note that the automated cells counting for DA_1_ (**E**) are not affected but DA_2_ (**F**) is significantly increased due to ERβ deficiency. Data are expressed as a box-and-whiskers plots, with the "box" depicting the median and the 25th and 75th quartiles, and "whiskers" showing 5th and 95th percentile. ** (*p* ≤ 0.01) and *** (*p* ≤ 0.001) indicates statistically significant differences between the studied groups of mice (ERβ^−/−^ and ERβ^+/+^ mice) analysed by Student's t-test. Elements—immunoreactive somata, fibres and neuropil. LA—lateral nucleus, BL—basolateral nucleus, BM—basomedial nucleus, CO—cortical nucleus, ME—medial nucleus and CE—central nucleus.

**Figure 2:**
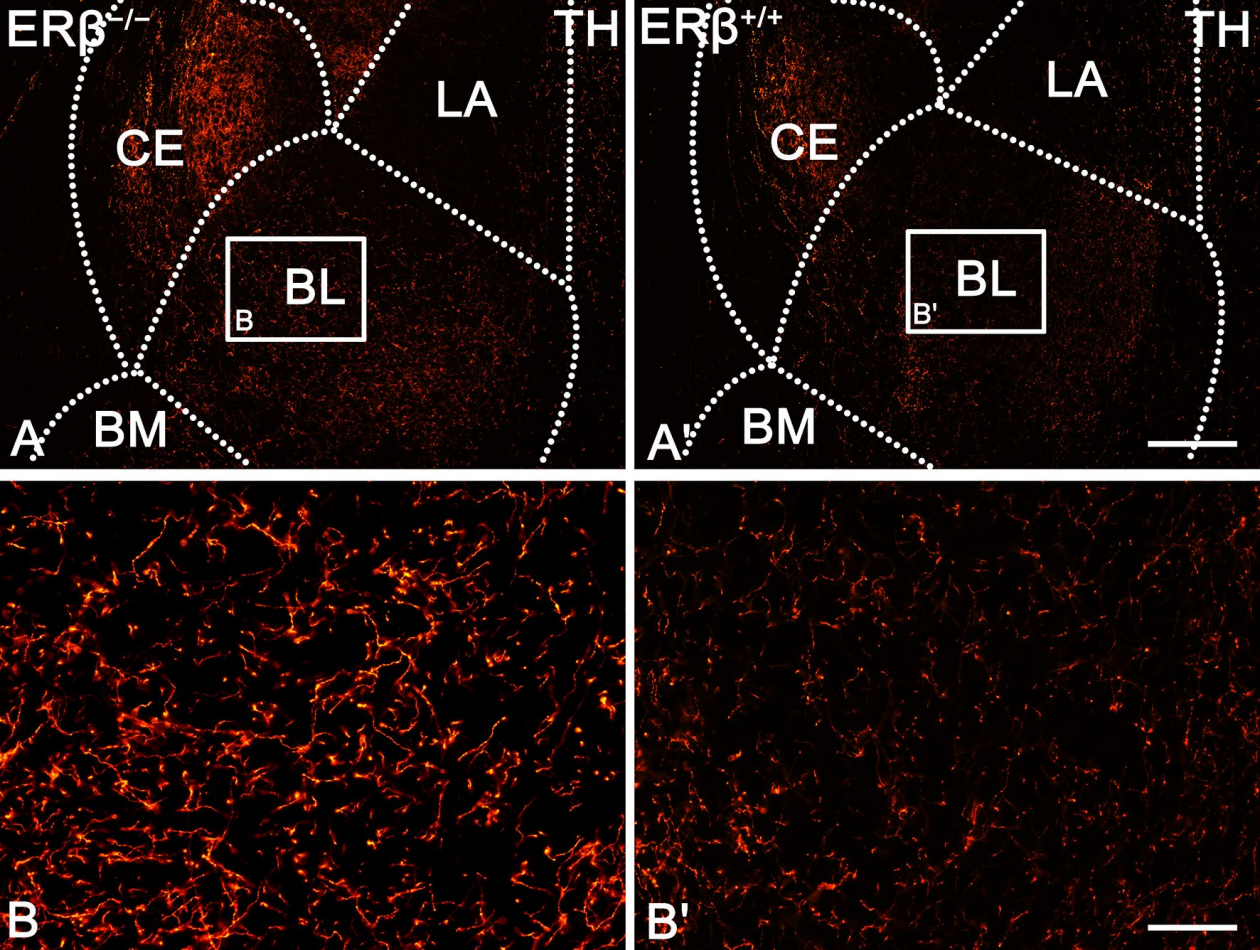
Representative colour photomicrographs illustrating the staining pattern of tyrosine hydroxylase (TH) in the amygdala of ERβ knock-out (ERβ^−/−^) and wild-type (ERβ^+/+^) mice, *n* = 6 per group. (**A**,**B**) ERβ^−/−^ mice. (**A′**,**B′**) ERβ^+/+^ mice. Note increased density and signal intensity of TH in ERβ^−/−^ mice (**A**,**B**) when compared to ERβ^+/+^ littermates (**A′**,**B′**). LA—lateral nucleus, BL—basolateral nucleus, BM—basomedial nucleus and CE—central nucleus. The scale bar applies to all microphotographs, and it corresponds to the length of 200 µm in (**A**,**A′**), and 50 µm in (**B**,**B′**).

**Figure 3:**
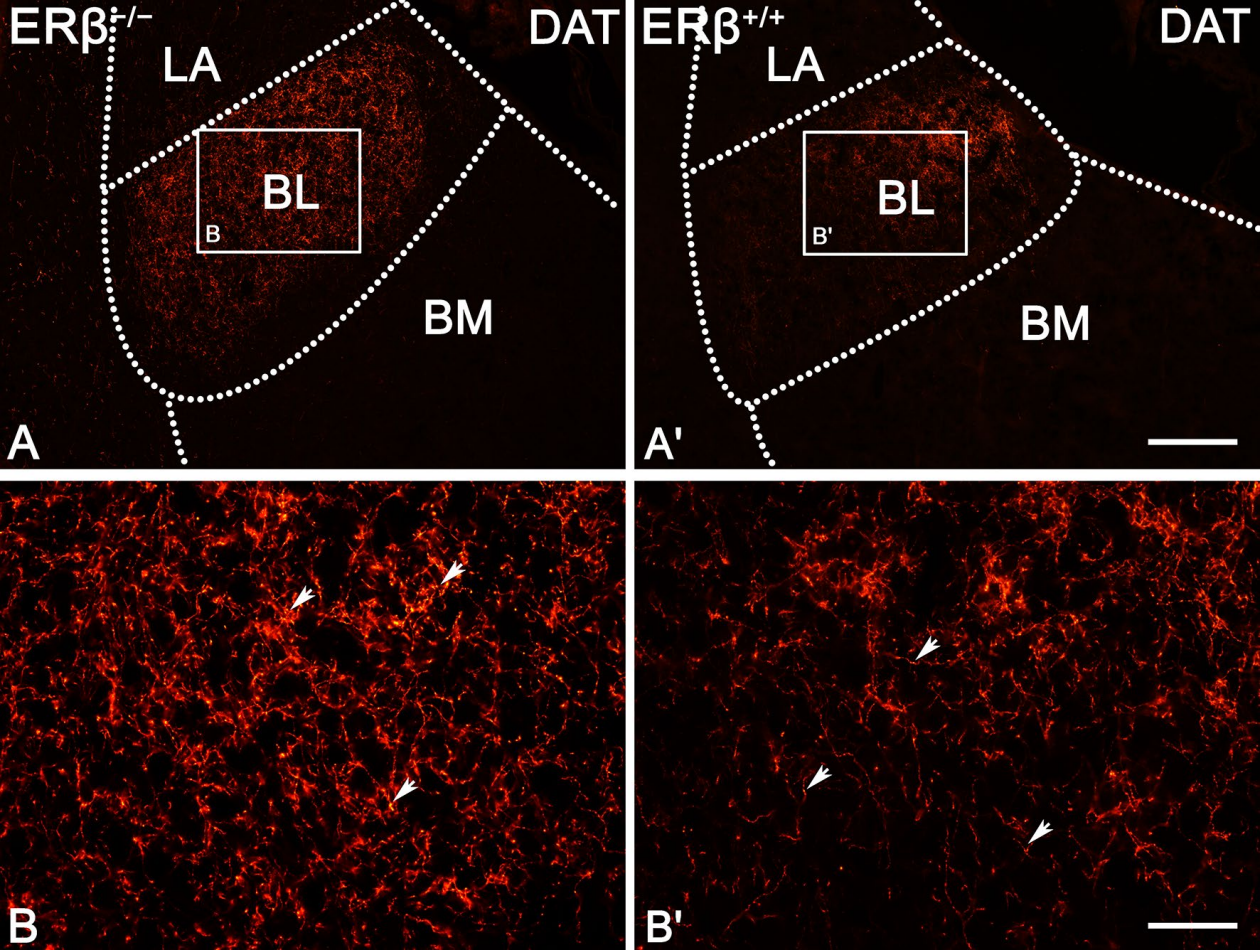
Representative colour photomicrographs illustrating the staining pattern of dopamine transporter (DAT) in the amygdala of ERβ knock-out (ERβ^−/−^) and wild-type (ERβ^+/+^) mice, *n* = 6 per group. (**A**,**B**) ERβ^−/−^ mice. (**A′**,**B′**) ERβ^+/+^ mice. Note increased density and signal intensity of DAT in ERβ^−/−^ mice (**A**,**B**) when compared to ERβ^+/+^ littermates (**A′**,**B′**). The arrows indicate areas where fiber swellings are located. LA—lateral nucleus, BL—basolateral nucleus, BM—basomedial nucleus. The scale bar applies to all microphotographs, and it corresponds to the length of 200 µm in (**A**,**A′**), and 50 µm in (**B**,**B′**).

Both, the characteristics and distribution of TH (Figs. 1A and 2) immunoreactivity were comparable in ERβ^−/−^ and ERβ^+/+^ mice, similar for the characteristics and distribution of DAT (Figs. 1B and 3), and they were consistent with previous results in the rodent amygdala^37,68–70^. For example, as in the mouse and rat, the immunoreactivity of TH (Fig. 2) and DAT (Fig. 3) were observed in fibres and puncta (Figs. 2A–B′ and 3A–B′). Furthermore, in both, ERβ^−/−^ and ERβ^+/+^ mice, the density of TH+ elements was very high in the CE, high in the BL, and from moderate to low in the rest of the amygdala (Figs. 1A and 2A,A′). In the case of DAT, a high density of immunoreactive elements was observed in the CE, moderate in the BL, while in the remaining nuclei it was at a very low level (Figs. 1B and 3A,A′). Detailed densitometric studies revealed that the volume density of TH+ and DAT+ elements was heavily elevated in all amygdala regions of ERβ^−/−^ mice when compared to matched controls (Fig. 1A,B and Table 1). The increase in TH density was generally in the range of 32–35%. The only exception was the CE with a value of ~ 25%. The increase in DAT density ranged from 28 to 30%, except for the LA with a value of ~ 25%.

**Table 1:** Statistical analysis of studied markers between ERβ^−/−^ and ERβ^+/+^ mice in the amygdala.

	LA		BL		BM		ME		CE		CO	
		p		p		p		p		p		p
TH	t_-7.45_ = 9.40	0.000031	t_-15.21_ = 6.52	0.000003	t_-10.99_ = 5.65	0.000050	t_-19.55_ = 8.62	0.000000	t_-17.07_ = 8.98	0.000000	t_-7.00_ = 7.30	0.000175
DAT	t_-12.64_ = 9.88	0.000000	t_-10.21_ = 9.36	0.000002	t_-8.97_ = 9.56	0.000005	t_-6.72_ = 6.42	0.0004	t_-21.87_ = 8.73	0.000000	t_-4.05_ = 7.14	0.005
DA_1_	t_10_ = − 0.28	0.79	t_10_ = − 0.43	0.67	t_10_ = 0.07	0.95	t_10_ = 0.19	0.85	t_10_ = 0.23	0.82	t_10_ = − 0.10	0.92
DA_1_ IR-neurons	t_10_ = − 1.08	0.30	t_10_ = 1.31	0.22	t_10_ = 1.35	0.21	t_10_ = − 1.68	0.12	t_10_ = 0.03	0.98	t_10_ = − 0.02	0.98
DA_2_	t_6.72_ = − 5.22	0.001386	t_7.39_ = − 9.85	0.000016	t_8.02_ = − 3.61	0.006820	t_9.64_ = − 5.67	0.000236	t_9.79_ = − 7.27	0.00003	t_6.13_ = − 5.07	0.002144
DA_2_ IR-neurons	t_9.83_ = − 8.87	0.000005	t_7.62_ = − 8.44	0.000039	t_7.40_ = − 10.74	0.000009	t_8.01_ = − 12.40	0.000002	t_8.24_ = − 9.00	0.000015	t_7.70_ = − 8.68	0.00003
ACHE	t_10_ = − 0.02	0.98	t_10_ = − 0.23	0.82	t_10_ = 0.73	0.48	t_10_ = 0.44	0.67	t_10_ = − 0.43	0.68	t_10_ = − 0.44	0.67
VAChT	t_10_ = − 0.14	0.89	t_10_ = 0.06	0.95	t_10_ = − 0.24	0.81	t_10_ = − 0.29	0.78	t_10_ = 0.46	0.66	t_10_ = 0.45	0.66
ACHR_M1_	t_9.34_ = − 11.11	0.000001	t_5.69_ = − 4.76	0.004	t_9.34_ = − 16.23	0.000000	t_9.10_ = − 12.72	0.000000	t_9.40_ = − 10.65	0.000002	t_7.52_ = − 11.81	0.000004
ACHR_M1_ IR-neurons	t_10_ = − 0.42	0.68	t_10_ = 0.49	0.64	t_10_ = − 0.44	0.67	t_10_ = 0.16	0.88	t_10_ = − 0.55	0.60	t_10_ = 0.31	0.77
ACHR_a7_	t_9.34_ = − 11.11	0.000001	t_5.69_ = − 4.76	0.004	t_9.34_ = − 16.23	0.000000	t_9.10_ = − 12.72	0.000000	t_9.40_ = − 10.65	0.000002	t_7.52_ = − 11.81	0.000004
ACHR_a7_ IR-neurons	t_10_ = 0.66	0.52	t_10_ = − 1.49	0.17	t_10_ = 1.10	0.30	t_10_ = 0.41	0.69	t_10_ = − 0.86	0.41	t_10_ = 0.66	0.52

*IR* immunoreactive.

**Figure 4:**
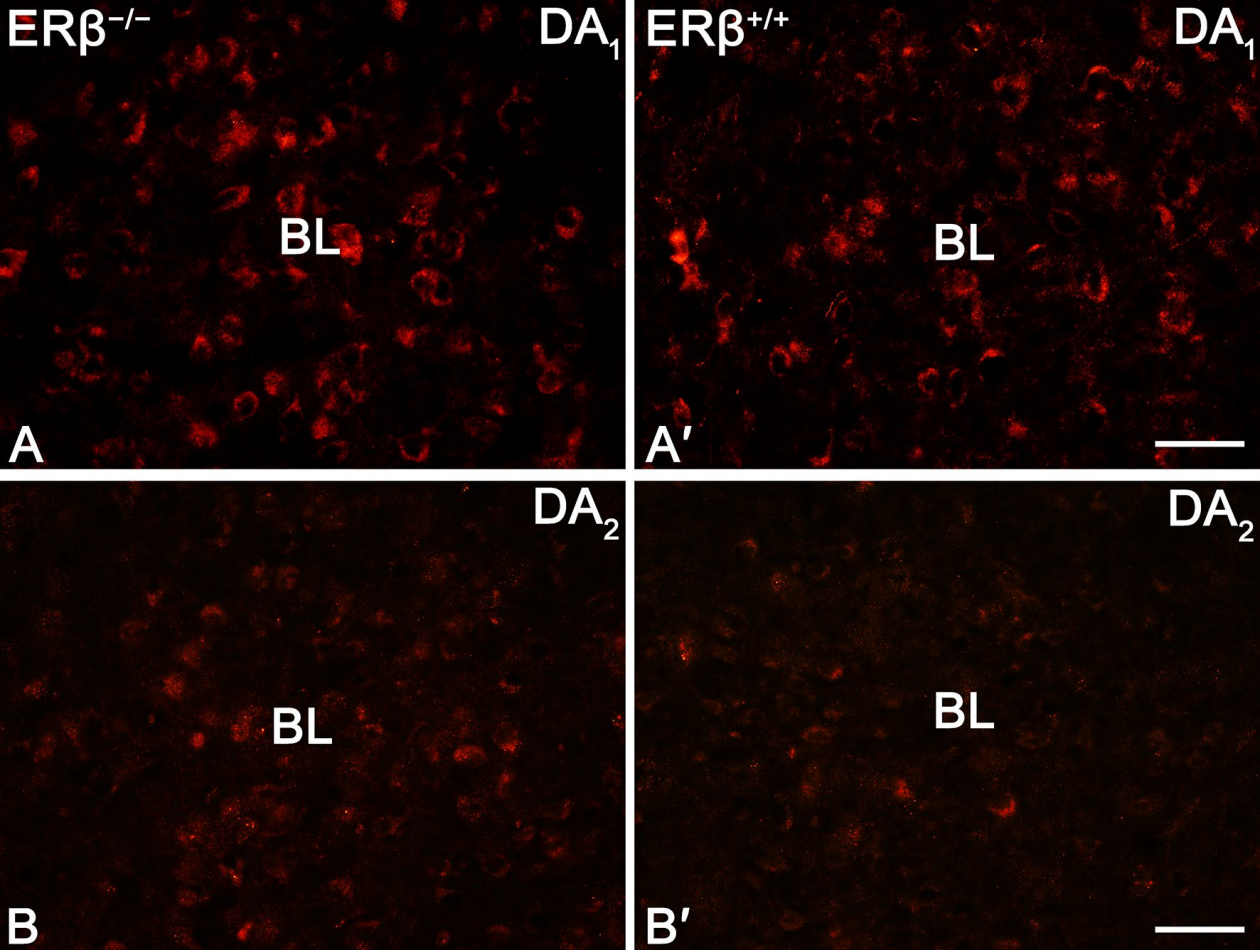
Representative colour photomicrographs illustrating the staining patterns of dopamine D_1_-like receptor (DA_1_) and dopamine D_2_-like receptor (DA_2_) in the amygdala basolateral nucleus (BL) of ERβ knock-out (ERβ^−/−^) and wild-type (ERβ^+/+^) mice, *n* = 6 per group. (**A**,**B**) ERβ^−/−^ mice. (**A′**,**B′**) ERβ^+/+^ mice. Note a lack of differences in DA_1_ density between ERβ^−/−^ mice (**A**) and ERβ^+/+^ littermates (**A′**). Note also increased density of DA_2_ in ERβ^−/−^ mice (**B**) when compared to ERβ^+/+^ littermates (**B′**). The scale bar applies to all microphotographs, and it corresponds to the length of 50 µm.

As was observed in previous studies in the rodent amygdala ^15,49,71,72^ in both mouse lines, DA_1_+ and DA_2_+ signal was mainly observed in soma and immunoreactive puncta, although some clearly depicted fibers were also present (Fig. 4). The density of DA_1_+ elements was heterogenous in the amygdala, and the highest it was in the BL, while in the CE it was the lowest (Fig. 1C). In the case of DA_2_, the highest density of immunoreactive elements was observed in the BL, whereas in the BM it was the lowest (Fig. 1D). Although the statistical analysis showed no significant differences in the volume density of DA_1_+ elements between ERβ knock-out and wild-type mice (Fig. 1C and Table 1), the volume density of DA_2_+ elements was elevated in the mutant mice (Fig. 1D and Table 1). Furthermore, higher number of immunoreactive neurons was present in DA_1_ than DA_2_ preparations (Fig. 1E,F). The DA_2_+ density increase was in the range of 11–13%. It is worth mentioning that apart from densitometric analyses, the neurons endowed with DA_1_ and DA_2_ were also additionally counted. This analysis revealed that the number of DA_1_+ neurons did not differ between ERβ knock-out (Fig. 4A) and wild-type mice (Figs. 1E, 4A′ and Table 1) while the number of DA_2_+ cells was elevated in mutant mice (Figs. 1F, 4B and Table 1).

### Cholinergic markers in the amygdala of ERβ knock-out mice

**Figure 5:**
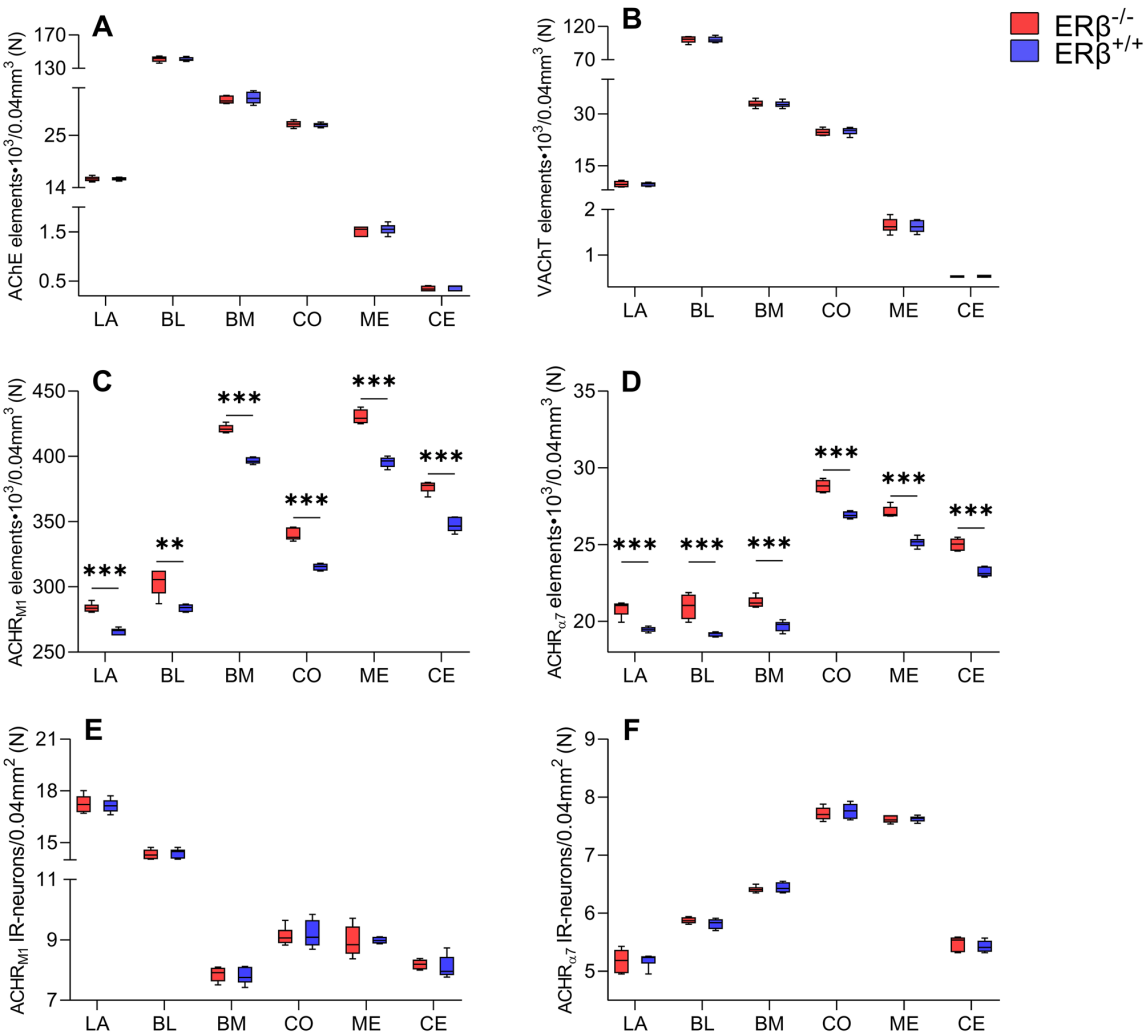
The densitometry of cholinergic markers such as acetylcholinesterase (AChE), vesicular acetylcholine transporter (VAChT), muscarinic acetylcholine type 1 receptor (AChR_M1_) and alpha-7 nicotinic acetylcholine receptor (AChR_α7_) in the amygdala of ERβ knock-out (ERβ^−/−^) and wild-type (ERβ^+/+^) mice, *n* = 6 per group. Note that the volume density of AChE (**A**) and VAChT (**B**) do not differ in both mice lines, but for AChR_M1_ (**C**) and AChR_α7_ (**D**) is significantly elevated in ERβ^−/−^ mice when compared to ERβ^+/+^ mice. Additionally, note that the automated cells counting for AChR_M1_ (**E**) and AChR_α7_ (**F**) are not affected due to ERβ deficiency. Data are expressed as a box-and-whiskers plots, with the "box" depicting the median and the 25th and 75th quartiles, and "whiskers" showing 5th and 95th percentile. ** (*p* ≤ 0.01) and *** (*p* ≤ 0.001) indicates statistically significant differences between the studied groups of mice (ERβ^−/−^ and ERβ^+/+^ mice) analysed by Student's t-test. Elements—immunoreactive somata, fibres and neuropil. LA—lateral nucleus, BL—basolateral nucleus, BM—basomedial nucleus, CO—cortical nucleus, ME—medial nucleus and CE—central nucleus.

**Figure 6:**
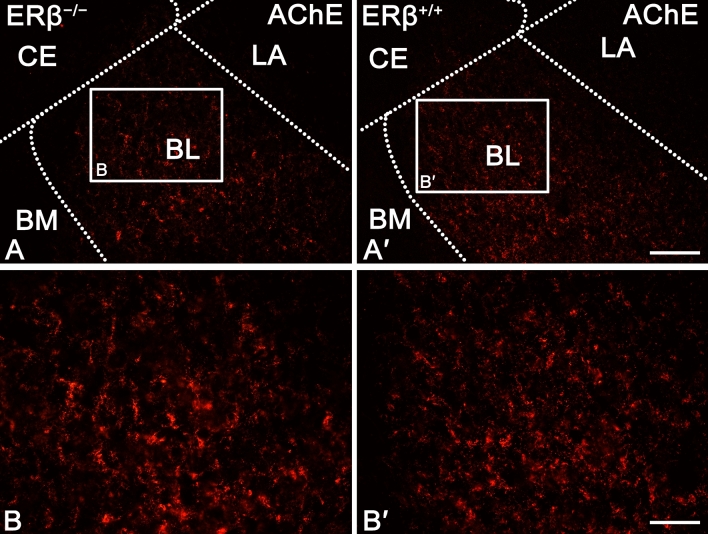
Representative colour photomicrographs illustrating the staining pattern of acetylcholinesterase (AChE) in the amygdala of ERβ knock-out (ERβ^−/−^) and wild-type (ERβ^+/+^) mice, *n* = 6 per group. (**A**,**B**) ERβ^−/−^ mice. (**A′**,**B′**) ERβ^+/+^ mice. Note similar density and signal intensity of AChE in ERβ^−/−^ (**A**,**B**) and ERβ^+/+^ mice (**A′**,**B′**). LA—lateral nucleus, BL—basolateral nucleus, BM—basomedial nucleus and CE—central nucleus. The scale bar applies to all microphotographs, and it corresponds to the length of 100 µm in (**A**,**A′**), and 50 µm in (**B**,**B′**).

**Figure 7:**
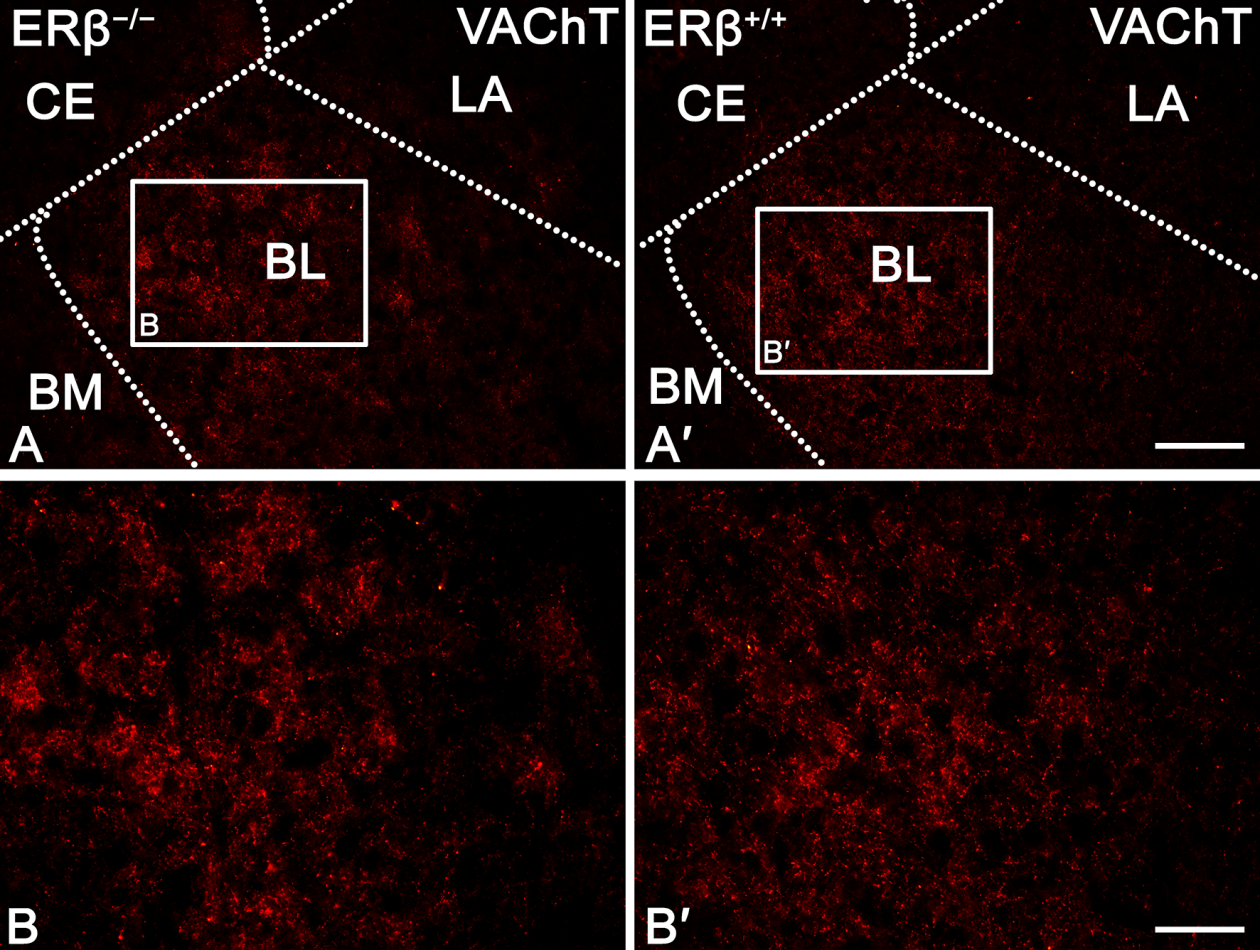
Representative colour photomicrographs illustrating the staining pattern of vesicular acetylcholine transporter (VAChT) in the amygdala of ERβ knock-out (ERβ^−/−^) and wild-type (ERβ^+/+^) mice, *n* = 6 per group. (**A**,**B**) ERβ^−/−^ mice. (**A′**,**B′**) ERβ^+/+^ mice. Note similar density and signal intensity of VAChT in ERβ^−/−^ (**A**,**B**) and ERβ^+/+^ mice (**A′**,**B′**). LA—lateral nucleus, BL—basolateral nucleus, BM—basomedial nucleus and CE—central nucleus. The scale bar applies to all microphotographs, and it corresponds to the length of 100 µm in (**A**,**A′**), and 50 µm in (**B**,**B′**).

The staining patterns for AChE (Figs. 5A and 6) and VAChT (Figs. 5B and 7) were quite similar in ERβ^−/−^ and ERβ^+/+^ mice, and they did not differ from the observations reported in the adult mouse and rat brain ^69,73–76^. For example, the immunoreactivity of both AChE (Fig. 6) and VAChT (Fig. 7) was present in fibres and puncta, and many of these fibres were thin and characterised by swellings (Figs. 6A–B′ and 7A–B′). However, in AChE preparations, the fibres were tightly packed while those in VAChT preparations were much less densely arranged (Figs. 6A–B′ and 7A–B′). Furthermore, in both, ERβ^−/−^ and ERβ^+/+^ mice, the density of AChE+ and VAChT+ elements was high in the BL, moderate in the LA, BM and CO, while in the ME and CE it was at a quite low level (Figs. 5A,B, 6A,A′ and 7A,A′). Finally, all these similarities in the staining patterns were additionally confirmed by densitometric analysis, which showed no significant differences in the volume density of AChE+ and VAChT+ elements between ERβ^−/−^ and ERβ^+/+^ mice (Fig. 5A,B and Table 1).

**Figure 8:**
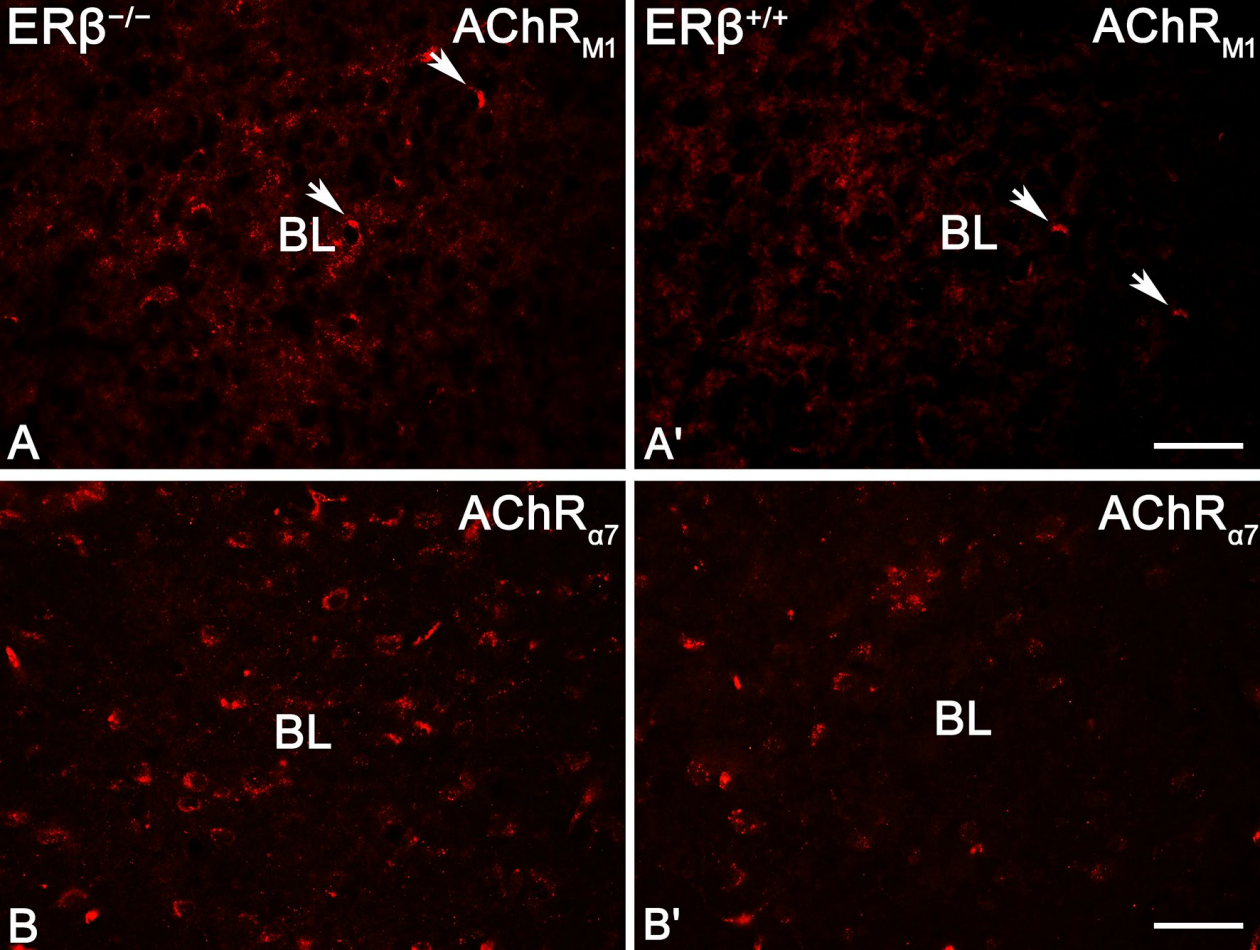
Representative colour photomicrographs illustrating the staining patterns of muscarinic acetylcholine type 1 receptor (AChR_M1_) and alpha-7 nicotinic acetylcholine receptor (AChR_α7_) in the amygdala basolateral nucleus (BL) of ERβ knock-out (ERβ^−/−^) and wild-type (ERβ^+/+^) mice, *n* = 6 per group. (**A**,**B**) ERβ^−/−^ mice. (**A′**,**B′**) ERβ^+/+^ mice. Note increased density of AChR_M1_ and AChR_α7_ in ERβ^−/−^ mice (**A**,**B**) when compared to ERβ^+/+^ littermates (**A′**,**B′**). Note also strong neuropil staining in AChR_M1_ preparations (**A**,**A′**) which makes it difficult to discern cells (arrows) from the background. The scale bar applies to all microphotographs, and it corresponds to the length of 50 µm.

AChR_M1_ and AChR_α7_ (Fig. 8) immunoreactivity was much more abundant in neuropil (immunoreactive puncta) than in DA_1_+ and DA_2_+ preparations, however, neuronal somata endowed with these proteins were also frequently present in both ERβ^−/−^ and ERβ^+/+^ mice. Similar characteristics were also previously described in some reports on the rodent amygdala^15,77–79^. It is worth noting that, AChR_M1_ immunoreactivity was much higher than the immunoreactivity for AChR_α7_ (Figs. 5C,D and 8). However, individual AChR_M1_+ (Fig. 8A,A′) perikarya were quite difficult to discern, as the level of immunoreactivity in neurons was often equal to that of the surrounding neuropil. On the other hand, on the surface of AChR_α7_+ (Fig. 8B,B′) perikarya there were frequently densely placed punctate structures. These punctate structures were also present within the neuropil, which was less strongly stained than in AChR_M1_ preparations. The densitometric studies showed that the density of AChR_M1_+ immunoreactive elements was the highest in the ME and BM, while the LA and BL showed the lowest density (Fig. 5C). For AChR_α7_, the ME and CO had the highest density while the LA, BL and BM had the lowest (Fig. 5D). These studies also revealed that the volume density of AChR_M1_+ and AChR_α7_+ immunoreactive elements was significantly elevated in all amygdala regions of ERβ^−/−^ mice when compared to matched controls (Fig. 5C,D and Table 1). The density increase was in the range of 7–8% for both AChR_M1_ and AChR_α7_. Additionally, a slightly higher number of immunoreactive neurons was present in AChR_M1_ than in AChR_α7_ preparations (Figs. 5E,F and 8). Interestingly, the automated counting of neurons endowed with AChR_M1_ and AChR_α7_ revealed that the number of the cells did not differ between ERβ^−/−^ and ERβ^+/+^ mice (Fig. 5E,F and Table 1).

### Protein–protein interaction network analysis

**Figure 9:**
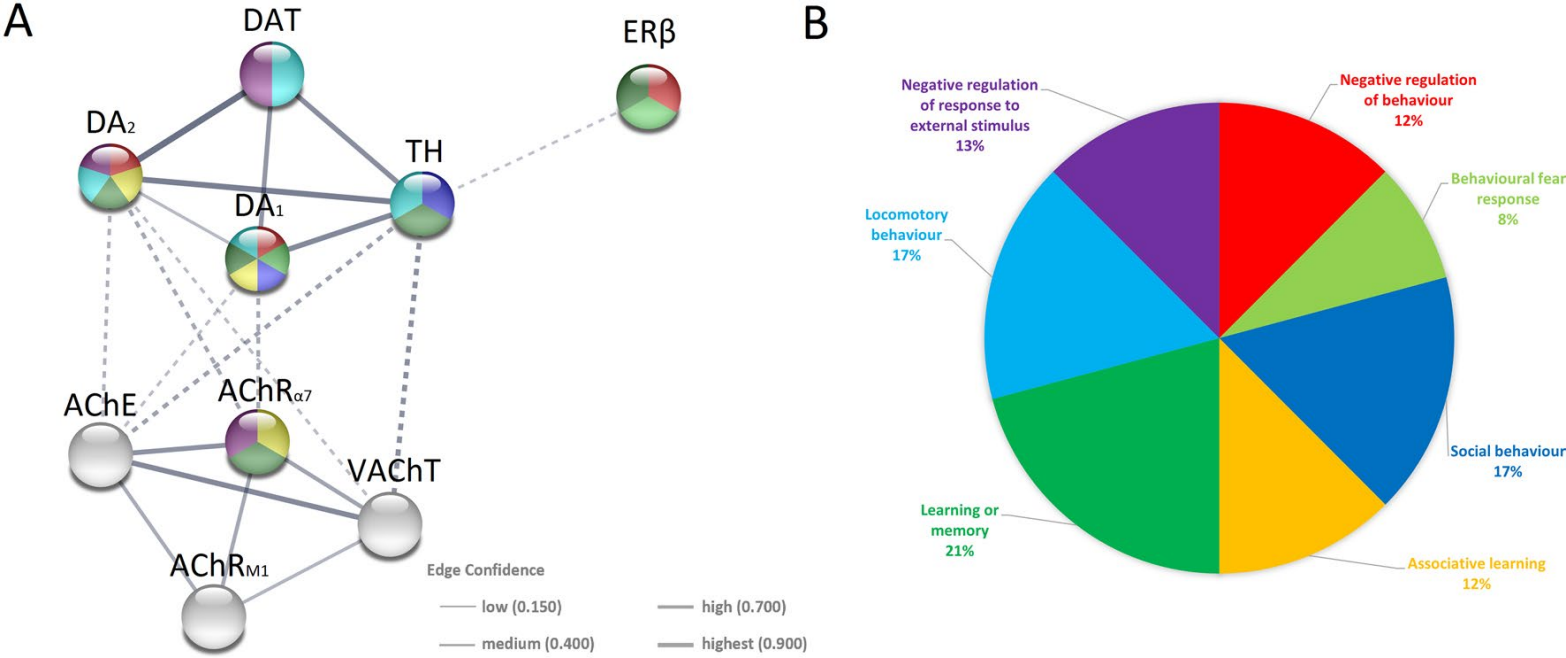
Protein–protein interaction (PPI) networks of potential targets by STRING database (*Mus musculus*). (**A**) PPI network of 9 potential targets, contains 9 nodes (proteins) and 20 edges (protein–protein associations). Dotted lines represent edges among 3 clusters (dopaminergic, cholinergic, and oestrogenic). The thickness of the line is proportional to the edge confidence. (**B**) Pie chart of term associated with emotional behaviour. Each colour represents a particular biological process; please note that studied markers are involved in these processes (**A**).

The protein–protein interaction (PPI) network contained 9 nodes and 20 edges, each node represented a target (dopaminergic, cholinergic and ERβ markers), each edge represented correlation evidence between 2 targets, and edges of different width represented different types of confidence from the low 0.150 to the highest 0.900. Disconnected nodes were not found, indicating there were direct or indirect interactions among the 9 potential targets with 129 biological processes (BP) significantly enriched, only 7 important BP connected with emotional behaviour processing were selected (*p* < 0.05, Table 2, Fig. 9). The entire analysis detected 5784 reference publications (PubMed), 3 clusters of local networks (STRING) and 3 Reactome pathways.

**Table 2:** Network statistics and biological process (Gene Ontology) of the interactions.

Network statistics			
Number of nodes	9		
Number of edges	20		
Average node degree	4.44		
Avg. local clustering coefficient	0.767		
Expected number of edges	1		
PPI enrichment *p*-value	< 1.0 × 10^–16^		

Biological process (gene ontology)	Description	Strength	*p*-value
Negative regulation of behaviour	Any process that stops, prevents, or reduces the frequency, rate	2.4	0.00014***
Behavioural fear response	An acute behavioural change resulting from a perceived external threat	2.08	0.0110*
Social behaviour	Behaviour directed towards society or taking place between members of the same species. Occurs predominantly, or only, in individuals that are part of a group	1.95	0.0171*
Associative learning	Learning by associating a stimulus (the cause) with a particular outcome (the effect)	1.84	0.0017**
Learning or memory	The acquisition and processing of information and/or the storage and retrieval of this information over time	1.61	0.0000675***
Locomotory behaviour	The specific movement from place to place of an organism in response to external or internal stimuli. Locomotion of a whole organism in a manner dependent upon some combination of that organism's internal state and external conditions	1.61	0.00059***
Negative regulation of response to external stimulus	Any process that stops, prevents, or reduces the frequency, rate, or extent of a response to an external stimulus	1.31	0.0233*

*(*p* ≤ 0.05), **(*p* ≤ 0.01) and ***(*p* ≤ 0.001) indicates statistically significant.

## Discussion

This is the first study to describe changes in the expression of main markers of dopaminergic and cholinergic systems in the amygdala of female mice lacking ERβ which exhibit increased anxiety in relation to their wild-type counterparts as reported in many papers^7,52,80^. The studies showed that among dopaminergic markers the expression of TH, DAT and DA_2_ was significantly increased in the amygdala of mice with ERβ deprivation in relation to matched controls whereas an ERβ deficiency did not affect DA_1_ expression. Analysis of the expression of cholinergic markers showed that, AChR_M1_ and AChR_α7_ were overexpressed, while the content of AChE and VAChT remain unaffected. The available data from various studies suggest that increased levels of TH, DA_2_ and AChR_M1_ may be an anxiolytic mechanism to reduce anxiety, whereas DAT overexpression appears to be anxiogenic. AChR_α7_ overexpression can both reduce and promote anxiety, so the role of AChR_α7_ is not obvious.

ERβ is considered as a potent anxiolytic and antidepressant factor^55^. It was reported that in female mice lacking ERβ, anxiety and depressed mood are also accompanied by alternations in neurotransmission and there is a strong link between oestrogen signalling and dopaminergic signalling^54,55^. Oestrogens modulate the activity of the catecholaminergic systems^53^ through increasing or decreasing DA activity, e.g., by its degradation, reuptake, and recover, also by upregulating dopaminergic^27^ or by reducing the affinity of the DAT^50^. Furthermore, many of these effects, have been shown to be mediated by ERβ signalling^51–55^. Our results showed that TH, DAT and DA_2_ expression was significantly increased in the amygdala of female ERβ deficient mice, suggesting a role for ERβ in monoamine neurotransmission and anxiety behaviour in mice and corresponds well with the results of other authors. Namely, stimulating as well as inhibiting effects of oestrogen on dopaminergic neurotransmission have been documented in the caudate putamen of ERβ knock-out mice^52^. Thus, in the absence of functional ERβ, regardless of the presence or absence of circulating oestradiol (E_2_) in plasma, female mice exhibited enhanced anxiety and decreased concentrations of DA and serotonin (5-HT) in the caudate putamen in comparison to the WT animals^52^. On the other hand, after E_2_ treatment in the culture, TH activity in the medial basal hypothalamus as well as DA content in the diencephalic but not in the mesencephalic from both adult male and female rats were reduced^81^. Finally, Jacome et al.^82^ found that ERβ activation altered the levels of DA’s metabolite (homovanillic acid, HVA) in several areas of the brain, including the prefrontal cortex, ventral hippocampus, and dentate gyrus, but not in the striatum or medial septum. To summarize, oestrogens appear to modulate DA neurotransmission in a manner that is region-specific.

The present analysis of dopaminergic markers showed that the expression of TH, DAT, and DA_2_ was significantly elevated in the amygdala of female mice lacking ERβ and which show increased anxiety-like behaviour compared to matched controls, while the expression of DA_1_ remains unchanged in these animals. This is consistent with the fact that increased TH and DAT levels were observed in young infant rats in anxiogenic conditions^83^, and DAT itself was significantly higher in the amygdala of patients with social anxiety disorder^18^. Interestingly, the DAT content in the amygdala of these patients positively correlated with the severity of symptoms^18^. Importantly, the loss of TH in the mice basolateral amygdala induces anxiety-like behaviour^17^ whereas in humans, increasing catecholamine levels (via tyrosine administration) before fear conditioning reduces fear expression^84^. Actually, tyrosine itself did not reduce anxiety, but rather, it weakened the responses of fear expression^84^. On the other hand, studies in mice with DAT knockout demonstrate that reduced DAT levels, which is one of the most important proteins engaged in dopaminergic tone regulation^85^, correlate with intensified turnover of DA and increased DA content in the synaptic cleft^86^. Mice with DAT deprivation also exhibit less anxiety in the elevated plus maze test and other anxiety-related paradigms^87^. These studies were in line with the earlier evidence indicating that exposure of mice to bisphenol A (BPA) during development disrupts the hormonal balance, and reduces expression of DAT and anxiety-like behaviours^88^. Oestrogens may up- or down-regulate DAT expression by both direct and indirect (kinase-mediated) interactions. Subsequently, these changes in DAT expression lead to modifications in the ability of the neurons to transport DA^89^. Interestingly, neurodegenerative diseases often appear first in women in life stages when physiological oestrogen levels start to fluctuate and then drop^90^. Thus, it seems that oestrogens that which previously maintained synaptic DA concentration by promoting outflow cease to regulate this process, e.g., in the case of ERβ deficiency^89^. The level of DA in the synapse thus falls, and then might promote the development of diseases characterized by low DA stimulation^89^. It should also be kept in mind that clear gender differences have been observed in dopaminergic neurotransmission, i.e. in the mesocortical organization^91,92^, which may have functional consequences for DA signalling processes. For example, sex differences and different endocrine states in women can alter response to DA medications, which has been well documented^90,93^.

It is worth noting that intracellular DA accumulation may result in oxidative stress and neurotoxicity, and then subsequently negatively affect the survival of neurons^94,95^. Furthermore, moderately intensified activity of the DAT leads to spontaneous loss of dopaminergic neurons that may be reversed by L-3,4 dihydroxyphenylalanine (L-DOPA, as a precursor to DA) application, suggesting that the integrity of DA neurons is mainly dependent on the DAT potential to maintain proper presynaptic DA homeostasis^96^. Interestingly, in 2-year-old ERβ knock-out female mice there is a remarkable amount of neurodegeneration of dopaminergic neurons, particularly in the substantia nigra^97^. Elevated DA_2_ content in the amygdala of ERβ knock-out subjects coincides with elevated expression of DA_2_ (short and long) isoforms in mice characterized by anxiety-like behaviours and depression^19^. Moreover, the dopaminergic mechanisms mediated by DA_2_ receptors are predominantly associated with fear expression rather than with fear acquisition^98^. Although, the mechanisms underlying the elevated DA_2_ content in the amygdala of ERβ knock-out mice is as yet obscure, but, as evidenced, conditioned fear intensifies DA release in the amygdala^99^ which may be reflected in the present study by TH up-regulation. Further, in response to the DA_2_ agonist, levels of fear-potentiated startle and freezing decreased, whereas in response to DA_2_ antagonist they increased^98,100^. On the other hand, assuming that released DA more probably will bind to DA_1_, which are more numerous in the amygdala than DA_2_—present study and Rouge-Pont et al.^101^, it could result in overactivation of adenylyl cyclase. Subsequently, the elevated levels of DA_2_ may be induced to manage with the overactivation of adenylyl cyclase as DA_2_ activation down-regulates adenylyl cyclase^43^. Interestingly, a low level of DA would predominantly activate DA_1_ receptors, disinhibiting and facilitating the amygdala function, whereas higher DA levels would also activate DA_2_ receptors^102^. A similar situation was also observed in the present study, as TH and DA_2_ were significantly elevated in the amygdala of mice with the lack of ERβ, whereas simultaneously no change in DA_1_ expression was observed in these animals. Taken together, these data suggest that elevated levels of TH and DA_2_ provide increased DA content and facilitate DA_2_ recruitment in the amygdala. Such conditions may constitute a kind of anxiolytic mechanism to reduce fear and anxiety-like behaviour. In contrast, DAT overexpression in the amygdala decreases DA availability in the synaptic cleft, promoting anxiety.

Amygdala cholinergic modulation in female mice with ERβ knock-out is altered. This observation corresponds with results of other authors, which reported that oestrogens play an important role in the cholinergic neurotransmission. For instance, ovariectomy decreased the high affinity choline uptake as well as choline acetyltransferase activity^103–105^, whereas oestrogen replacement therapy reversed these effects^105,106^. In addition, an increase, and a decrease of the AChR_M_s density in the hypothalamus and in the preoptic area, respectively, in oestrogen-treated ovariectomized rats, when compared with ovariectomized rats have been shown^107^. In the rat hippocampus, ovariectomy up-regulated AChR_M4_^108,109^ and the E_2_ treatment reversed this effect^108^. Furthermore, adult female rats, showed greater variation in density and affinity of AChR_M_s in cortical tissues than male rats^110^. Thus, interaction between oestrogens and cholinergic system shows discrepancies that could be the result of species related differences, sex differences, and might be associated with brain areas and hormonal status of the animals, resulting in differential expression of cholinergic markers.

There is evidence indicating that AChR_α7_ is involved in circuits controlling mood and anxiety, when activated, can induce both anxiolytic^111^ and anxiogenic effects^112^. For example, studies in rats proved that in the basolateral amygdala, AChR_α7_ increase primarily GABAergic inhibitory tone, and when they are further exogenously activated they enhance spontaneous inhibitory postsynaptic currents in glutamatergic excitatory neurons, significantly diminishing overall excitability of these cells^79^. This may be one of the potential pathways allowing nicotine to suppress excitability of the amygdala and in this way reduce anxiety^113^ and alleviate depression^114^. This also suggests that the elevated content of AChR_α7_ in the amygdala of ERβ knock-out mice could be a mechanism to counteract increased anxiety-like behaviour in these animals. On the other hand, mecamylamine infusion into the mouse amygdala (non-competitive and non-selective nicotinic receptors’ antagonist), viral-mediated downregulation of the AChR_α7_ or knock-out AChR_α7_ all resulted in strong anxiolytic- and antidepressant-like effects^61^. Moreover, negative allosteric modulation of AChR_α7_ via BNC210 decreases reactivity of the amygdala in patients with generalized anxiety disorder^115^, while the amygdala’s hyperactivity to stimuli related to threat is a hallmark of anxiety^116^. These phenomena may be explained by the fact that AChR_α7_ are present not only in inhibitory but also in excitatory neurons^15,79^, and can enhance both GABAergic^79^ and glutamatergic^117,118^ neurotransmission in the amygdala. Furthermore, AChR_α7_ are especially crucial for synaptic plasticity in this brain region^119^. Interestingly, rats with anxiety disorder due to traumatic brain injury have significant surface expression of AChR_α7_ and current mediated by these receptors on principal neurons which are one of the important contributors of anxiety^120^. Considering that oestrogens reduce neuronal excitability in the amygdala^13^ through their receptors^14^, thereby reduced oestrogen signalling as a result of ERβ knock-out would be anxiogenic. Thus, AChR_α7_ antagonism can mediate an anxiolytic- and antidepressant-like response, and increased expression of these proteins in the ERβ knock-out mice could be one factor in increasing anxiety-like behaviour. Taken together, overexpression of AChR_α7_ in ERβ knock-out female mice may reflect two opposite mechanisms, and which of these is actually present in these animals requires further study.

Increased expression of AChR_M1_ in the amygdala of mice deprived of ERβ is quite prominent as AChR_M1_ are mostly present on projection neurons^78^ while there is a significant loss among these cells in ERβ knock-out mice^24^. Although AChR_M1_ are not directly linked with the expression of anxiety-like behaviours, they are, however, involved in several types of emotional/motivational learning including contextual fear conditioning and fear extinction^121^; and impaired fear extinction is a hallmark of anxiety-related disorders^122^. The reduced expression of AChR_M1_ within the amygdala was correlated with deficits in fear extinction learning, however, the increase in AChR_M1_ expression in this brain region may be related to intact and properly functioning fear extinction learning^123^. Further, direct injection of the muscarinic cholinergic agonist oxotremerine into the basolateral amygdala elicited an enhancement in fear extinction learning^63^. In addition, systemic AChR_M1_ antagonism reduced contextual fear extinction, while positive allosteric modulation of AChR_M1_ increased consolidation of contextual fear extinction in mouse model of posttraumatic stress disorder^124^. Finally, analyses using cevimeline (an AChR_M1_ agonist) and telenzepine (an AChR_M1_ antagonist), as well as AChR_M1_ knock-out mice showed that AChR_M1_ regulates the cued fear memory consolidation by redundant activation of phospholipase C in the basolateral amygdala^125^. Taken together, AChR_M1_ are required for fear extinction mediated by the amygdala, and overexpression of these proteins in mice with increased anxiety and cellular deficits may be a compensatory mechanism to alleviate fear extinction deficits and reduce anxiety.

The STRING database generated a protein–protein interaction (PPI) network for all proteins tested in this study and showed strong interactions between them. Moreover, the Gene Ontology (GO) molecular function enrichment analysis identified seven terms (biological processes) associated with behaviour and cognitive functions of *Mus musculus*, influenced by ERβ, as well as by the dopaminergic and cholinergic markers studied. Based on the conducted STRING of PPI analysis, it can be assumed that the changes in behaviour^7^ and cognitive functions impairments^126^ observed in ERβ^−/−^ female mice may be related to the changes in dopaminergic and cholinergic transmission observed in the present study. This assumption needs to be verified by experimental data, especially since the available literature lacks any data on this subject.

It should be kept in mind that future studies are required to elucidate in detail the exact role of these markers in the formation of anxiety. For example, measurements of DA and ACh concentrations as well as the content of other markers in the amygdala by Western blot, ELISA and/or HPLC quantifications would probably allow for more precise functional conclusions.

## Materials and methods

All details information about materials and small equipment were added to Supplementary 1.

### The animals

Adult female mice, i.e., ERβ^−/−^ (homozygous B6.129P2-Esr2^tm1Unc^/J, common name: ERβ KO, *n* = 6, aged 6–8 weeks) and ERβ^+/+^ (C57BL/6J, common name: B6, *n* = 6, aged 6–8 weeks) were provided by the Jackson Laboratory (Bar Harbor, ME, USA). The number of animals for this study was estimated based on a pilot study using studied markers in 3 brains in each group (*n* = 3 /ERβ^−/−^and *n* = 3 /ERβ^+/+^). This study indicated that the individual differences were small, the increase in the number of n did not significantly affect the obtained results, and *n* = 6 was sufficient. After acclimatization for 1 week housed in the animal facilities at the Faculty of Veterinary Medicine of the University of Warmia and Mazury (Olsztyn, Poland) under standardized conditions: 21 ± 1 °C, 12–20 air exchanges/h, 12-h shift of the light–dark cycle, free access to feedstuff devoid of phytoestrogens (LabDiet^®^ JL Rat and Mouse/Auto 6F 5K52) and tap water (ad libitum). To avoid stress from isolation, animals were kept separately in sanitized polypropylene cages in a group of three. The care and handling of animals was strictly in line with the European Union Directive for animal experiments (2010/63/EU) and principle of the 3Rs: replacement, reduction, and refinement. Thus, all animals used were registered, and the staff was properly trained (Certificate No 1267/2015). This study is reported in accordance with ARRIVE guidelines. All efforts were made to minimize animal suffering and use the minimum number of animals necessary to generate reliable scientific data. It is worth noting that the strains of mice used in the present study were selected cautiously. We chose to use ERβ^−/−^ females rather than ERβ^−/−^ males, as ERβ^−/−^ females are the best validated animal model of reduced oestrogen signalling and anxiety disorders based on genetic, behavioural, and neurobiological studies^7,52,80^. For example, results of anxiety-related tests such as the open field and the elevated plus maze (routinely used tests to study anxiety-related behaviour in mice) conducted in female and male ERβ^−/−^, and their wild-type (WT) control mice showed significant genotype differences and consistent behavioural abnormalities that are sensitive to enhanced anxiety. In the open field test, ERβ^−/−^ females showed a significant increase in thigmotaxis and latency to move from the centre of the field to the wall comparing to both WT control and male ERβ^−/−^ mice. On the other hand, total locomotion was significantly reduced in these mice^7,127,128^. Furthermore, in the elevated plus maze test, ERβ^−/−^ mice spent less time in the open arm compared to other study groups^7^. The C57BL/6J mice were used to serve as a wild-type control according to the recommendations of the Jackson Laboratory (http://jaxmice.jax.org/strain/004745). The B6.129P2-Esr2tm1Unc/J mice were derived directly from C57BL/6J substrain mice (wild-type littermates), which prevented any genetic differences in the studied case.

### Anaesthesia and tissue processing

After a 2-week-long habituation phase, mice were exposed to the same laboratory procedures. Before anaesthesia, monitoring of the oestrous cycle was performed to determine the stage of the oestrous cycle based on vaginal smear^13,129^. To avoid the oestrogen-induced anxiolytic effect, all mice were anaesthetized in metaestrous; a phase associated with a decline in oestrogen level^130^. Intraperitoneal injection anaesthesia in mice was done with pentobarbital (Morbital, Biowet, Poland; 2 mL/kg) according to Humane Society Veterinary Medical Association guidelines, and each mouse, after cessation of breathing, was transcardially perfused with sodium chloride (0.9%) followed by 4% paraformaldehyde (PFA) diluted in 0.1 M phosphate-buffered saline (PBS). The brains were then removed and were fixed in 4% PFA for overnight, washed three times in 0.1 M PBS (pH 7.4, 4 °C) and then transferred (for 3–5 days) to sucrose in 1xPBS at 4 °C (10%, 20% and 30%) as cryoprotectant. Lastly, the brains were frozen and then cut coronally into 10 μm thick sections using a cryostat. The sections were mounted on glass slides and stored at − 80 °C until use.

### Immunohistochemistry

Representative sections of the amygdala from both ERβ^−/−^ and ERβ^+/+^ mice were stained using a routine two-way immunofluorescent procedure. The details of this procedure have been described previously by Równiak^131^. All immunostaining steps were performed using humid chambers and at room temperature (RT). Briefly, to visualize all markers, sections were triple-washed in PBS and then incubated for 1 h at RT with a blocking buffer (0.1 M PBS, 10% normal donkey serum, 0.1% bovine serum albumin, 0.05% thimerosal and 1% Tween-20). These tissue samples were then incubated with primary antibodies diluted in a blocking buffer at RT overnight (Table 3). After that, sections were rinsed three times in PBS and then incubated for 1 h with a solution of secondary antibodies (Table 3). Finally, slides were coverslipped in carbonate-buffered glycerol (pH 8.6).

**Table 3:** Specification of antigens.

Antigen	Code	Clonality	Host species	Dilution	Supplier	Location
Primary antibodies						
TH	Ab112	Polyclonal	Rabbit	1: 2000	Abcam	Cambridge/UK
DAT	Ab184451	Monoclonal	Rabbit	1: 1000	Abcam	Cambridge/UK
DA_1_	ADR-001	Polyclonal	Rabbit	1: 500	Alomone labs	Jerusalem/Israel
DA_2_	AB5084P	Polyclonal	Rabbit	1: 200	Millipore	Temecula, CA/USA
ACHE	Ab183591	Polyclonal	Rabbit	1: 500	Abcam	Cambridge/ UK
VAChT	PA5-85782	Polyclonal	Rabbit	1: 2000	Thermo Fisher	Rockford, IL/USA
AChR_M1_	AB5164	Polyclonal	Rabbit	1: 500	Millipore	Temecula, CA/USA
AChR_α7_	Ab216485	Polyclonal	Rabbit	1: 500	Abcam	Cambridge/UK
NeuN	ABN78	Polyclonal	Rabbit	1: 1000	Millipore	Temecula, CA/USA
Secondary antibodies						
ALEXA Fluor 555-nm	A-31572	Polyclonal	Donkey anti-rabbit	1: 800	Thermo Fisher	Rockford, IL/USA
ImmPRESS HRP Universal Antibody (Ig, Peroxidase)			Donkey anti-rabbit	1:1	Vector Laboratories	Burlingame, CA/USA

It is worth noting that to define the location and boundaries of individual amygdala nuclei in these sections, a series of sections from our previous study^24^ and from the same mice which were stained using antibodies directed against a neuron-specific nuclear protein (NeuN, pan-neuronal marker) were used. These sections (15 slides in space 70–80 μm) were subjected according to the immunoperoxidase labelling with 3.3-diaminobenzidine (DAB) as a substrate-chromogen. Briefly, these sections were preincubated for 30 min in 0.3% H_2_O_2_ diluted in 99.85% methanol and then blocked for 60 min with a solution of 10% normal donkey serum (diluted in PBS). After primary antibodies’ incubation, the sections were triple-washed in PBS, treated with the solution of peroxidase-conjugated secondary antibodies for one hour (Table 3), and finally incubated with a 3% DAB solution. Then, sections were dehydrated, cleaned in xylene and mounted in slide mounting medium, a mixture of distyrene, plasticizer, and xylene (DPX). After this, on all DAB-stained slices, the coordinates for individual amygdala nuclei were determined according to the method previously described by Sterrenburg et al.^132^.

### Controls

The specificity of the primary antisera used in this study was shown by various researchers using these products in multiple previous studies. Furthermore, all antibodies were positively validated by immunoblots (more in Supplementary 2). The specificity of the secondary antibodies was assessed by omitting the primary antibody or replacing it with non-immune sera or PBS. The specificity of the secondary antibody was confirmed by the lack of any immunosignal.

### Counts and measurements

The density of the dopaminergic and cholinergic markers in the amygdala nuclei, was quantified on immunofluorescence-labelled sections and analysed using an Olympus BX51 microscope (Olympus GmbH, Germany) equipped with a digital camera (CC-12, Soft Imaging System, Münster, Germany) and Cell-F software (Olympus, Hamburg, Germany). The lateral (LA), basolateral (BL), basomedial (BM), medial (ME), central (CE) and cortical (CO) nuclei were tested and the delineation pattern of these nuclei and the amygdala nomenclature have been adopted (without any modification) from recent version of rodent atlas of Paxinos and Franklin^74^. In each animal of both ERβ^−/−^ and ERβ^+/+^ mice, fifteen analyses for evenly spaced sections (per antigen and per nucleus) arranged from the rostral (bregma =  − 0.82) to the caudal extent (bregma =  − 2.46) of the amygdala^74^ were done. Additionally, density analyses performed in the present study (line scan analysis for volume density and automated cells counting) on the single section were always done with a 40× lens and with the use of 347.6 µm × 260.7 µm regions (test frames). These frames had an area of the computer screen, and they were always arranged to cover the full area of the analysed nucleus. Firstly, we measured the volume density of immunoreactive elements (somata, fibres, neuropil) according to the line scan analysis described and validated by Sathyanesan et al.^133^. Next, we measured in the same test frame immunoreactive neurons using automated cell counting. The density values calculated from individual test frames on a section were always reduced to the section average. As this average only referred to the area of the test frame, it had to always be converted to a value corresponding to the area of 0.04 mm^2^ or 0.04 mm^3^. To calculate antigen density in the particular nucleus, the results were averaged across all sections for each animal. Finally, the values obtained from each amygdala nucleus were also averaged to each group of mice and saved in the format: mean ± standard deviation (SD).

All analyses (volume density as well as manual and automated cell counting) were carried out on coded slides. These analyses were done by the principal investigator, and then they were repeated by two independent researchers in a blinded manner (ERβ^−/−^ and ERβ^+/+^ mice). The obtained results showed a high degree of inter-rater reliability (Pearson R = 0.79).

#### Volume density counting

To evaluate the volume density of TH, DAT, DA_1_ and DA_2_ as well as AChE, VAChT, AChR_M1_ and AChR_α7_ immunoreactive elements in the amygdala nuclei of ERβ knock-out and wild type mice, stained sections were analysed according to the automated line scan analysis described and validated by Sathyanesan et al.^133^. The same parameters were used for capturing the image, including exposure time and camera gain, which provide a good grayscale dynamic range for all images collected from different sections and animals. All images were saved in 8-bit TIF grayscale format. Mathematically, fluorescently labelled structures can be represented as curvilinear structures with local intensity variations (minima or maxima)^133^, these local intensity extremes can be detected using a Hessian matrix^134^. In this way, filtering the image based on the Hessian matrix extracts line-like information from the input image. Consequently, all analysed fluorescence images were extracted by the Hessian-based filter included in the plugin called FeatureJ^135^ for NIH ImageJ software (version 1.53e). As the plugin offers several parameters to be chosen from, according to validation by Sathyanesan et al.^133^ the following parameter options were selected: “largest eigenvalue of Hessian tensor” option, and “absolute eigenvalue comparison” option and set the “smoothing scale” factor to 0.5. Next, the evaluation of these images by line scan profile analysis was carried out using five lines oriented horizontally (parallel scans) and the same number of lines oriented vertically (perpendicular scans). Lines were drawn using ImageJ’s “line tool” through the region of interest (ROI). Either straight or segmented line were drawn depending on the morphology of the amygdala nuclei. In the following step, the line scans were baseline-adjusted and then processed with a peak detection algorithm. The estimation of the baseline is conditioned by the manner of distinguishing peaks (signal) from the background. To estimate the background noise for the line scans, these scans were always collected from several places of the section which characterized by absence of immune signal. Each mean was then calculated based on these measurements and constituted the ultimate background value for the single section (threshold) for a peak detection algorithm.

#### Manual and automated cells counting

Before taking an image, the number of DA_1_+, DA_2_+, AChR_M1_+ or AChR_α7_+ neurons with a clearly visible labelling in the cell soma was manually counted using a high-resolution microscope (Olympus GmbH, Germany). For automated cell counting, the number of cells was counted on previously captured images using the NIH ImageJ software (version 1.53e). In the first step, after selecting the "dark background" option, which separates cells from the dark background, the analysed image was always converted to a binary image in order to extract objects based on their circularity. Then the particle size was set with the parameters 1800–2500 pixels and the circularity discrimination with the parameters 0.2–1.0. The number of test frames for each nucleus was the same in both methods: LA: 1–4, BL: 2–6, BM: 2–3, CO: 1–2, ME: 4–6, and CE: 2–3.

### Protein–protein interaction (PPI) network

Additionally, the search tool for the retrieval of interacting genes (STRING) was used as an online tool for the protein–protein interaction (PPI) network analysis^67^. In this study, selected PPI networks of mice (*Mus musculus*) were constructed by STRING to access interactions among studied markers. The cut-off criterion *p* < 0.05 and Markov Clustering (MCL) of nodes were chosen. The results were filtered by the following criteria: p-adjusted ≤ 0.05 (Benjamini–Hochberg correction), query protein only, full STRING network, confidence of network edges, confidence equal to 0.400, and three group of clusters. Next, Gene Ontology (GO, Gene Ontology Resource) enrichment analysis was performed to visualize and indicate what biological functions the studied dopaminergic and cholinergic markers correspond to.

### Statistic

The present research used a mixed model, and the parametric method was utilized for the whole analysis. The calculations were made with the use of Statistica 13.3 software (TIBCO Software Inc., Palo Alto, CA, USA). Statistically significant (α = 0.05) differences between experimental and control groups (ERβ^−/−^ and ERβ^+/+^ mice) were detected by using the appropriate test statistics assuming the statistical power (1-β) was 0.95, and the allocation ratio was 1:1. Since the sample size of the ERβ^−/−^ and ERβ^+/+^ mice was six individuals per group, fifteen sections were analysed per animal. The data from immunohistochemical studies were averaged and then examined using Shapiro–Wilk tests. While an independent-sample t-test with multiple comparisons was used to determine characteristic differences between the two groups. The examined homogeneity of variance was used, and then the Cochran Q test correction was performed. The significance level was set at *p* ≤ 0.05. All statistical graphs presented in the study were made in GraphPad Prism 6 software (GraphPad Software, La Jolla, CA, USA). Data are presented with box-and-whisker plots. The “box” representing the median as well as 25th and 75th quartiles, and “whiskers” showing the 5th and 95th percentile (*n* = 6 per group).

### Ethical approval

This study is reported in accordance with ARRIVE guidelines.

## Conclusions

This study describes abnormalities in the expression of the main markers of the dopaminergic and cholinergic systems in the amygdala of female mice lacking ERβ, which are characterized by increased anxiety. The results show that the expression of TH, DAT, DA_2_, AChR_M1_, and AChR_α7_ is significantly elevated in the amygdala of mice with ERβ knock-out in relation to wild-type control subjects whereas the content of DA_1_, AChE, and VAChT is not affected by ERβ deficiency. Available data from multiple studies suggest that increased levels of TH, DA_2_, and AChR_M1_ may constitute an anxiolytic mechanism to alleviate anxiety, whereas DAT overexpression seems to be anxiogenic. The role of AChR_α7_ overexpression is not obvious, as increased levels of these proteins may reduce or promote anxiety.

## Supplementary Information

Supplementary Information 1.

Supplementary Information 2.

## Data Availability

The data that support the findings of this study are available from the corresponding author upon reasonable request.
