# Supplementary material for: Dopaminergic and cholinergic modulation of the amygdala is altered in female mice with oestrogen receptor β deprivation
Source: Sci Rep. 2023 Jan 17;13:897. doi: 10.1038/s41598-023-28069-2 (PMC9845293; doi:10.1038/s41598-023-28069-2)
Supplement: Supplementary file 1 — Supplementary Information 1. [file 41598_2023_28069_MOESM1_ESM.docx]

**Supplementary 1.** Details information about materials and small equipment

| Materials and small equipment | Code | Supplier | Location |  |  |  |
| --- | --- | --- | --- | --- | --- | --- |
|  |  |  |  |  |  |  |
| Bovine serum albumin | A7030 | Sigma Aldrich | Taufkirchen, Germany |  |  |  |
| Cryostat | HM525 | Zeiss | Oberkochen, Germany |  |  |  |
| DPX Mountain for histology | 44581 | Sigma Aldrich | Taufkirchen, Germany |  |  |  |
| Ethanol | 396480427 | POCH | Gliwice, Poland |  |  |  |
| Glycerol | G9012 | Sigma Aldrich | Taufkirchen, Germany |  |  |  |
| Hydrogen peroxide | 1.08597 | Sigma Aldrich | Taufkirchen, Germany |  |  |  |
| Immuno Slide Staining Trays | R64001-E | Pyramid Innovation Ltd. | Chaucer Business Park, UK |  |  |  |
| Methanol | 1424109 | Sigma Aldrich | Taufkirchen, Germany |  |  |  |
| Normal donkey serum | 17-000-121 | Jackson ImmunoResearch | Cambridge, UK |  |  |  |
| Paraformaldehyde pH 7.4 | 1040051000 | Sigma Aldrich | Taufkirchen, Germany |  |  |  |
| Phosphate buffer | P7994 | Sigma Aldrich | Taufkirchen, Germany |  |  |  |
| Phosphate-buffered saline | P5493 | Sigma Aldrich | Taufkirchen, Germany |  |  |  |
| Sodium chloride | 07982 | Sigma Aldrich | Taufkirchen, Germany |  |  |  |
| Sucrose | 363-117720907 | ALCHEM | Toruń, Poland |  |  |  |
| Thimerosal | T5125 | Sigma Aldrich | Taufkirchen, Germany |  |  |  |
| Tween-20 | 11332465001 | Sigma Aldrich | Taufkirchen, Germany |  |  |  |
| 3.3-diaminobenzidine | D7679 | Sigma Aldrich | Taufkirchen, Germany |  |  |  |
