# Supplementary material for: Dopaminergic and cholinergic modulation of the amygdala is altered in female mice with oestrogen receptor β deprivation
Source: Sci Rep. 2023 Jan 17;13:897. doi: 10.1038/s41598-023-28069-2 (PMC9845293; doi:10.1038/s41598-023-28069-2)
Supplement: Supplementary file 2 — Supplementary Information 2. [file 41598_2023_28069_MOESM2_ESM.docx]

**Supplementary 2.** Specificity of the antisera

| Antisera | Code | Host | Western blot analysis | Molecular mass | References |
| --- | --- | --- | --- | --- | --- |
|  |  |  |  |  |  |
| anti-TH | ab112 | rabbit | rat | 62 kDa | Boundy et al. 1993; Li et al. 2019; Williams et al. 2020; Liu et al. 2021 |
| anti-DAT | ab184451 | rabbit | mouse and rat | 69 kDa |  |
| anti-DA_1_ | ADR-001 | rabbit | mouse and rat | 75 kDa |  |
| anti-DA_2_ | AB5084P | rabbit | rat | 50 kDa |  |
|  |  |  |  |  |  |
| anti-ACHE | ab183591 | rabbit | mouse and rat | 68 kDa | manufacturer’s technical information and Levey et al. 1991; Dong et al. 2020 |
| anti-VAChT | PA5-77386 | rabbit | mouse and rat | 59 kDa |  |
| anti-AChR_M1_ | AB5164 | rabbit | rat | 70 kDa |  |
| anti-AChR_α7_ | ab216485 | rabbit | mouse | 56 kDa |  |
|  |  |  |  |  |  |
| anti-NeuN | ABN78 | rabbit | mouse | 48 kDa | Lo Iacono et al. 2015 |
|  |  |  |  |  |  |
